# NADA Ear Acupuncture and Medical Acupuncture for Pain- and Health-Related Quality of Life among Older Patients with Chronic Nonspecific Low Back Pain: A Prospective Clinical Trial

**DOI:** 10.3390/brainsci14030205

**Published:** 2024-02-23

**Authors:** Monika Rybicka, Jerzy Gąsowski, Anna Przeklasa-Muszyńska, Jan Dobrogowski, Jagoda Wierzbicka, Ka-Kit Hui, Sara Ptasnik, Magdalena Kocot-Kępska

**Affiliations:** 1 Department of Internal Medicine and Gerontology, Faculty of Medicine, Jagiellonian University Medical College, 31-008 Krakow, Poland; jerzy.gasowski@uj.edu.pl; 2 Department of Medicine, Center for East-West Medicine, David Geffen School of Medicine, University of California, Los Angeles, CA 90024, USA; khui@mednet.ucla.edu (K.-K.H.); sptasnik@mednet.ucla.edu (S.P.); 3 Department of Pain Research and Treatment, Faculty of Medicine, Jagiellonian University Medical College, 31-501 Krakow, Poland; a.przeklasa-muszynska@uj.edu.pl (A.P.-M.); jan.dobrogowski@uj.edu.pl (J.D.); jagodawierzbicka@gmail.com (J.W.)

**Keywords:** NADA protocol, medical acupuncture, chronic nonspecific low back pain, nonpharmacological pain management, older patients

## Abstract

Background: The purpose of this study was to investigate the efficacy and safety of the NADA (National Acupuncture Detoxification Association)-standardized ear acupuncture protocol in comparison to medical acupuncture (MA) in the treatment of chronic nonspecific low back pain (LBP) in older adults. Methods: This was a prospective, clinical, single center, open label, comparative study. A total of 60 older patients with chronic nonspecific LBP were enrolled in the study. The patients were divided into two groups. The MA group received treatment with medical acupuncture (MA), while the NADA group received NADA ear acupuncture once a day for 20 min, for a total of 10 sessions. The co-primary outcome measures were the reduction in pain intensity evaluated by the Numeric Rating Scale (NRS) compared to baseline and improvement in patients’ quality of life (QOL) assessed in the SF-36 questionnaire before and after treatment; this was compared between the two groups. Results: After two weeks of treatment, a significant reduction compared to baseline was observed in the NRS scores following treatment with medical acupuncture as well as after the utilization of NADA ear acupuncture protocol: NRS score for average pain experienced by the patients over the previous week (NRSa) MA: *p* = 0.002; NADA: *p* < 0.001, maximum NRS score in the past week (NRSm) MA: *p* < 0.001; NADA: *p* < 0.001, and NRS score at the time of examination (NRSe) MA: *p* = 0.001; NADA: *p* < 0.001. Reduction of the NRSa score compared to baseline was significantly greater in the NADA group (*p* = 0.034). Significant improvements in the QOL of patients according to the SF-36 questionnaire compared to baseline were observed in the MA group in the following domains: PF (*p* = 0.003), RP (*p* = 0.002), SF (*p* = 0.041), RE (*p* = 0.005), MH *(p* = 0.043), HT (*p* = 0.013), PCS (*p* = 0.004), and MCS (*p* = 0.025); and in the NADA group, in the following domains: PF (*p* = 0.004), RP (*p* = 0.048), BP (*p* = 0.001), VT (*p* = 0.035), RE (*p* = 0.006), MH (*p* < 0.001), HT (*p* = 0.003), PCS (*p* < 0.001), and MCS (*p* < 0.001). There were minor complications observed in 35% of patients (total of 20 participants); 31% (9 patients) in the MA group and 39% (11 patients) in the NADA group. These were minor and quickly resolved, including insertion point pain, minor bleeding after needle removal, and one instance of fainting. No patients in either group reported worsening of LBP. These complications occurred in 4.14% of MA sessions (12 times/290 sessions) and in 6.07% of NADA acupuncture sessions (16 times/280 sessions). Conclusion: The outcomes of this study suggest that both MA and NADA ear acupuncture could be a valuable and personalized component of a comprehensive approach to managing chronic nonspecific LBP in older patients. Incorporation of MA and NADA ear acupuncture into the clinical management of chronic nonspecific LBP in elderly patients has the potential to reduce pain intensity and improve the overall quality of life of affected individuals. However, further studies are needed to confirm our findings.

## 1. Introduction

Low back pain (LBP) is defined as pain or discomfort localized between the costal margin and inferior gluteal fold [1]. When the duration of such pain exceeds three months, it is classified as chronic [2]. Although the complete range of potential causes for LBP is extensive, most of them are rarely encountered in general medical practice [3,4]. In primary care settings, less than 1% of patients presenting with LBP will have a serious underlying cause, such as cauda equina syndrome, metastatic cancer, or spinal infection [3]. Structural abnormalities affecting the spine itself, such as compression fractures, spinal stenosis, and disk herniations, are more common, accounting for approximately 10 to 15% of cases [3]. Most patients seen in primary care (>85%) will exhibit nonspecific LBP, which refers to pain in the absence of a specific underlying condition that can be reliably identified [3]. Diagnosis of nonspecific low back pain is made after specific disorders of spinal and nonspinal origin (i.e., spinal stenosis, sciatica, tumor, inflammatory disease, fracture of the spine, pain due to pathology of internal organs) are ruled out [4]. This type of pain is often referred to as “idiopathic” LBP and is commonly associated with chronic or recurring symptoms [3]. From a mechanistic point of view, chronic nonspecific LBP is a mixed condition with nociceptive, neuropathic and nociplastic components involved [5].

LBP is a major cause of pain and disability around the world [6]. Among all chronic pain problems and spinal pain conditions, LBP is considered the most common public health, economic, and social problem [7]. According to sample interviews of the US civilian population in 2018, 31.6% of women and 28% of men aged 18 years or older reported having LBP in the past 3 months [8]. These numbers increase even more in the geriatric population. A recent systematic review including a total of 135,059 elderly individuals assessed the prevalence of LBP to be between 21–75% [7]. Managing chronic LBP, especially in older adults, poses challenges due to the presence of multiple medical conditions, which can lead to polypharmacy, increased risk of drug–drug and drug–disease interactions, and reduced drug tolerability, particularly in cases involving kidney or liver dysfunction. The over-reliance on medications and invasive procedures and surgeries may result in serious side effects, underscoring the need for longer-term solutions for pain management in this specific population. The effectiveness of drugs and interventional techniques in chronic nonspecific LBP is limited, therefore a multimodal treatment with nonpharmacological pain management techniques is recommended [6,9,10]. A recent systematic review, which synthesized evidence from European neck and LBP clinical practice guidelines, found inconsistent or inconclusive recommendations regarding the use of acupuncture for neck pain and LBP [11]. Recommendations from the European guidelines contrast with a systematic review of non-invasive treatments for LBP conducted to inform the American College of Physicians (ACP) Clinical Practice Guideline [10]. Recommendation 2 (with Grade: strong recommendation) of the ACP practice guideline stated that for patients with chronic LBP, nonpharmacological treatments should be initially selected, such as exercise, multidisciplinary rehabilitation, acupuncture, mindfulness-based stress reduction, tai chi, yoga, progressive relaxation, electromyography biofeedback, and cognitive behavioral therapy [10].

Acupuncture is a therapeutic intervention that involves the insertion of fine, solid, metallic needles into specific sites on or through the skin [3]. Over thousands of years, manual acupuncture has accumulated significant clinical use in China, and its practice has spread to Japan in the sixth century and then to Europe in the seventeenth century [12]. Today, it is widely practiced and approved in many countries and regions as an important treatment modality [10,12]. The main analgesic mechanism of action of acupuncture in Western medicine is thought to stimulate the antinociceptive-descending pathways to release several neurotransmitters and endogenous opioids [13]. Moreover, it may include local antidromic axon reflexes, segmental and extrasegmental neuromodulation [13]. Acupuncture can also affect the hypothalamic-pituitary-adrenal axis (HPA) to lower cyclooxygenase-2 (COX-2) and prostaglandin E2 (PGE2) levels and enhance the sympathetic nervous system to cause the peripheral release of opioids, exerting anti-inflammatory effects in these ways [13]. Acupuncture has been reported to inhibit sympathetic activity and modulate the endocrine system by downregulating the HPA axis [13].

Ear acupuncture, also known as auricular acupuncture, and nowadays named auriculotherapy, is a type of reflexive treatment which involves the stimulation of specific points on the ear that correspond to various organs and systems in the body [14]. Applying needles to these specific ear acupoints elicits a reflex response to affect physical, emotional, and neurological dysfunctions [14]. Ear acupuncture has garnered attention from the broader medical community in recent years with increased accessibility in various clinical settings [15]. One of the mechanisms of action of ear acupuncture is thought to be mediated via vagal nerve stimulation [16], but other mechanisms may be involved, including trigeminal nerve stimulation (TNS) [17]. The NADA protocol, also known as acudetox, is a widely implemented 5-point-standardized ear acupuncture protocol that was initially developed to address substance abuse, posttraumatic stress disorder (PTSD), depression, and anxiety [18,19]. It has also been used for pain management, smoking cessation, and to improve general wellbeing [18]. Several of the acupoints used in the NADA protocol include the ear points for the “Lung”, “Liver”, and “Kidney”, all of which are found in the concha region of the auricle that is innervated by the parasympathetic vagus nerve. The NADA protocol also includes the nearby “Sympathetic” point, the functionally opposing part of the autonomic nervous system (ANS) [16].

There have been several studies on acupuncture effectiveness in LBP in recent years. Referring to Urtis I. et al. (2021), despite their heterogeneity in the core outcome measurement sets used, many of them show promising results supporting the use of acupuncture in patients with LBP. Authors of this study concluded that acupuncture can be a first-line treatment for patients suffering from low back pain [12].

Reevaluation of Systematic Reviews and Meta-analyses published in 2023 aimed to reassess the methodological quality, report quality, and evidence quality of 23 systematic reviews (SRs)/meta-analyses (MAs) of acupuncture for LBP to determine whether acupuncture can effectively treat LBP [20]. According to this study, acupuncture was more effective than placebo, Western medicine, sham acupuncture, physical therapy, usual care, or Transcutaneous Electrical Nerve Stimulation (TENS) in treating LBP [20]. What is more, acupuncture combined with other treatments was more effective than acupuncture alone in relieving LBP [20]; although the methodological, report, and evidence-based quality of the SRs/MAs on acupuncture for LBP was described as low [20]. Authors suggested that the results of all existing SRs/MAs may overestimate the actual effects of acupuncture and further studies with an improved methodological design are needed to accurately determine the actual effectiveness and safety of acupuncture in the management of LBP [20].

A large-scale systematic review and meta-analysis of randomized placebo and sham-controlled trials of 2110 patients from 1980 to 2016 published in 2020 assessed the evidence for the efficacy of acupuncture for nonspecific LBP, compared with sham or placebo therapies. Pain intensity and disability were measured using the Visual Analogue Scale (VAS) and the Roland Morris Disability Questionnaire (RMDQ), respectively. The study concluded that, in the treatment of subacute and chronic nonspecific LBP, there was moderate evidence for the efficacy of acupuncture as a short-term treatment method, with few adverse effects for decreasing pain intensity when compared to sham or placebo acupuncture [21].

Authors of another recent systematic review from 2023 were uncertain whether acupuncture was more effective and safer than conventional therapy for chronic nonspecific LBP [22]. The study concluded that there was low or very low certainty evidence for the effectiveness of acupuncture in decreasing pain intensity or disability in comparison to nonpharmacological or pharmacological treatment [22]. When compared to combined pharmacological and non-pharmacological treatment, authors found moderately certain evidence that acupuncture reduced pain intensity, and low-quality evidence that it reduced disability in patients with chronic, nonspecific LBP [22]. Authors found very low certainty evidence for the effectiveness of acupuncture as an add-on to non-pharmacological treatment in reducing pain and disability [22]. When acupuncture was prescribed as an add-on to combined pharmacological and non-pharmacological treatment, authors found no difference in reducing pain, but an improvement in disability, with very low certainty evidence [22].

Limited data currently exist regarding the use of ear acupuncture according to the NADA protocol or a simple standardized protocol of medical acupuncture (MA) in the elderly population experiencing chronic nonspecific LBP. This study aimed to compare the effectiveness of MA and NADA ear acupuncture as nonpharmacologic pain management options in older patients with chronic, nonspecific LBP.

## 2. Materials and Methods

### 2.1. Study Design

This was a clinical, prospective, single-center, open-label, comparative study conducted at the Department of Pain Research and Treatment of the Jagiellonian University Medical College in Krakow, Poland. The study was conducted according to the guidelines of the Declaration of Helsinki and approved by the Biomedical Ethics Commission of the Jagiellonian University in Krakow, Poland (No. of approval: 1072.6120.7.2021, date: 20 January 2021). The informed consent process was meticulously carried out in accordance with the ethical guidelines and principles set forth by the Biomedical Ethics Commission of the Jagiellonian University in Krakow, Poland. Informed consent was obtained from all subjects involved in the study, after receiving a comprehensive explanation of the study’s objectives, procedures, potential risks, and benefits. The document was provided to them in their native language and in a format that was easily comprehensible, ensuring that they had ample opportunity to seek clarifications and ask questions. The consent form and the entire process were approved by the Biomedical Ethics Commission of the Jagiellonian University in Krakow, Poland to guarantee the protection of participants’ rights and welfare.

**Figure 1:**
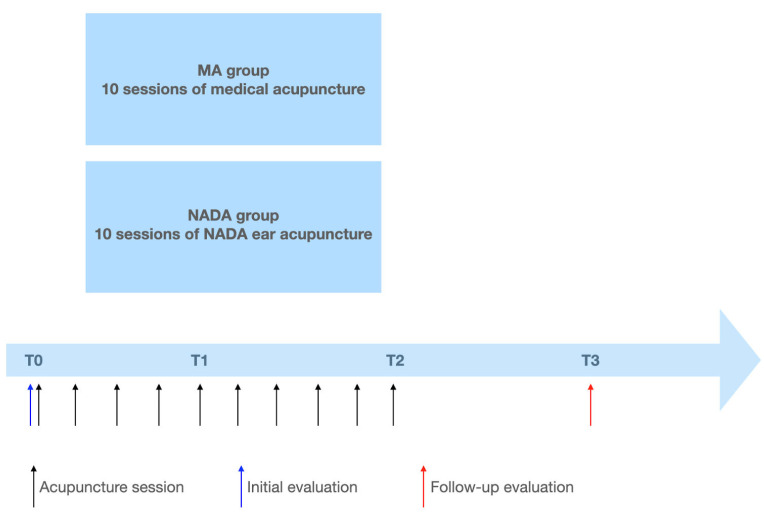
Study timeline: T0: initial visit and pre-treatment evaluation; T1: 1 week after beginning of acupuncture sessions; T2: 2 weeks after beginning of acupuncture sessions, end of treatment; T3: final visit 3 weeks after beginning of acupuncture sessions, evaluation one week after completion of treatment; MA: Medical acupuncture; NADA: National Acupuncture Detoxification Association.

The study was conducted from January 2021 to August 2022 and included older patients (age greater than or equal to 60) diagnosed with chronic nonspecific LBP according to ACTTION-APS Pain Taxonomy (AAPT) criteria [23]. A total of 60 older patients with chronic nonspecific LBP were enrolled in the study. The patients were divided into two groups. The group assignment procedure involved two distinct stages. Initially, individuals who were about to begin their MA treatment sessions, prescribed as a part of a comprehensive multimodal pain treatment plan by a pain specialist, were approached to ask about their interest in participating in the study. Upon their agreement and confirmation of meeting the inclusion criteria, they were subsequently enrolled in the MA group. On the other hand, patients on the waiting list for MA treatment were extended invitations to join the study as a part of the NADA group. The MA group received treatment with medical acupuncture, while the NADA group received NADA ear acupuncture once a day for 20 min, for a total of 10 sessions. Data were collected at the initial evaluation prior to treatment and again one week after treatments had concluded (see Figure 1). The co-primary outcome measures were the reduction in pain intensity evaluated by the Numeric Rating Scale (NRS) compared to baseline, and improvement in patients’ quality of life (QOL) assessed in the SF-36 questionnaire before and after treatment, and this was compared between the two groups. Additionally, proportions of patients achieving 30% and 50% reduction in average NRS pain scores over the previous week were assessed.

There were three NRS scores assessed in the study: NRS score for average pain experienced by the patients over the previous week (NRSa), maximum NRS score in the past week (NRSm), and NRS score at the time of examination (NRSe). The study assessed the patients’ quality of life (QOL) before and after treatment using the SF-36 questionnaire. As a rescue treatment, paracetamol 500 mg per dose was advised with a maximum daily dose of 6 tablets per day (if no contraindications). Patients were asked to carefully monitor and record the number of tablets consumed each day throughout the study duration and report it at the follow-up evaluation. The treatment plan for the patients was established during the regular visit by a pain specialist at the Department of Pain Research and Treatment of the Jagiellonian University Medical College in Krakow, Poland. The pain was treated adequately to the patient’s needs, evidence-based knowledge, and concomitant diseases. As the patients were suffering from chronic pain and had already tried most pharmacologic treatment options, they decided for nonpharmacologic pain management. At the time of inclusion to this study, their treatment of chronic LBP was only short-term oral medications such as NSAIDs, metamizole, or tramadol/paracetamol. Once the patients entered the study, they were asked to use oral paracetamol when needed.

### 2.2. Patients

During the study period, eligible participants were identified by experienced pain specialists through the screening of consecutive patients seeking medical consultation at the Department of Pain Research and Treatment of the Jagiellonian University Medical College in Krakow, Poland. The study population consisted entirely of individuals of Caucasian ethnicity, with 100% of the participants identifying as Caucasian. This demographic homogeneity was a result of regional demographics. The inclusion criteria for the study were as follows: age greater than or equal to 60 years, a diagnosis of chronic nonspecific LBP made by a pain specialist from the Department of Pain Research and Treatment according to the ACTTION-APS Pain Taxonomy (AAPT) criteria [23] for pain duration of at least 3 months (chronic pain), pain classified as moderate or severe with an intensity rating of 3 or higher on the NRS, the patient’s voluntary and informed consent to participate in the study after a clear presentation of the associated risks and benefits of the treatment, the patient’s ability to comprehend the content of the scales and questionnaires, absence of contraindications for acupuncture, and no history of acupuncture treatment within the previous 3 months.

Exclusion criteria from the study encompassed the following: lack of patient consent, presence of acute or decompensated advanced diseases, pregnancy, patient’s inability to understand and comply with instructions, advanced psychiatric and neurological disorders, coagulation disorders, generalized inflammatory skin diseases, and infectious diseases.

**Figure 2:**
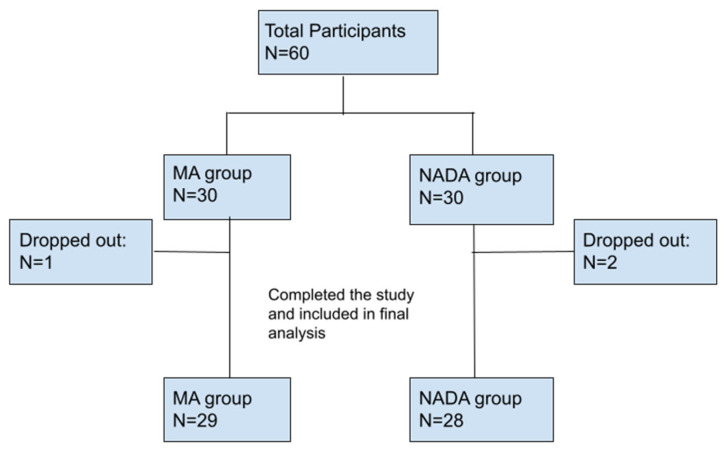
Number of participants in each study group: MA: Medical acupuncture; NADA: National Acupuncture Detoxification Association; N: number of patients.

In the MA group, one participant dropped out of the study and did not attend the follow-up visit without providing an explanation. In the NADA group, two participants discontinued their involvement in the study: one participant reported difficulties with clinic access, while another participant did not attend the follow-up visit without providing a reason (see Figure 2).

### 2.3. Interventions

Prior to the commencement of acupuncture treatment, patients underwent a thorough examination conducted by a medical doctor from the Department of Pain Research and Treatment of the Jagiellonian University Medical College in Krakow, Poland. Patients also completed initial data collection prior to receiving acupuncture treatment (T0).

**Figure 3:**
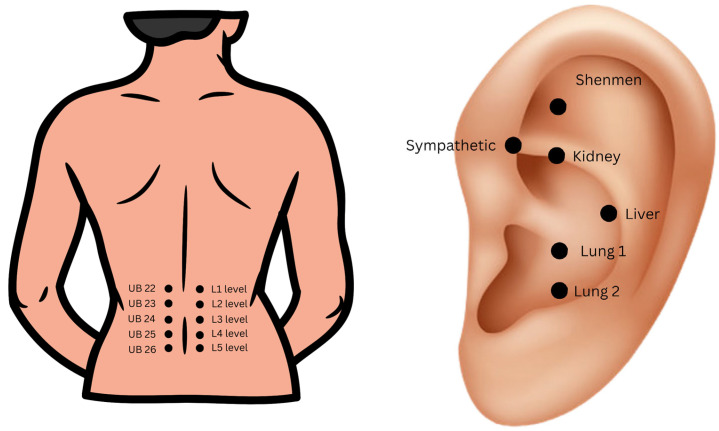
Acupoints used in the MA and NADA groups; MA: Medical acupuncture; UB: Urinary Bladder; L: the level of lumbar vertebra; NADA: National Acupuncture Detoxification Association. Pictures of ear and back prepared in the program Canva (https://www.canva.com) (accessed on 15 July 2023).

The acupuncture procedures were performed by a medical doctor who had received training in this technique. In the MA group, needles were applied locally in the low back area. The standard protocol involved placing the needles along the spine at points corresponding to the Chinese acupuncture points bilaterally on the Urinary Bladder (UB) meridian: UB 22, UB 23, UB 24, UB 25, and UB 26 (see Figure 3). These points were chosen as they correspond locally to common areas of tenderness in chronic nonspecific LBP and are located 1.5 cun (Chinese measurement: the width of the two forefingers [24]) lateral to the back midline on the level of L1 to L5 vertebras respectively. In this study, standard needles commonly utilized for treatment at the pain department were used, measuring 0.30 mm × 30 mm. Treatment sessions were conducted once daily for a duration of 20 min each, totaling ten days excluding Saturdays and Sundays (T0–T2). In the NADA group, standardized acupoints were utilized according to the NADA protocol, which includes “Shenmen”, “Sympathetic”, “Kidney”, “Liver”, and “Lung” (see Figure 3) [18]. As the “Lung” point has two possible locations, “Lung 1” and “Lung 2”, the practitioner examined each point and selected the more tender point for treatment. Unilateral, alternating treatments were administered, with the left ear needled on one day and the right ear on the other, alternating between sessions. For ear acupuncture, standardized needles measuring 0.16 mm × 15 mm were used. As in the MA group, treatment sessions were conducted once daily for a total of ten days excluding Saturdays and Sundays (T0–T2). The needles remained in the patient’s ear for a duration of 20 min. One week (T3) after the final acupuncture session, patients underwent a follow-up assessment (see Figure 1).

### 2.4. Outcomes

The co-primary outcome measures were the reduction in pain intensity evaluated by the Numeric Rating Scale (NRS) compared to baseline (T0) and improvement in patients’ quality of life (QOL) assessed in the SF-36 questionnaire before (T0) and after treatment (T3), and this was compared between the two groups. We assessed the NRS score for average pain experienced by the patients over the previous week (NRSa), maximum NRS score (NRSm), and NRS score at the time of examination (NRSe). Furthermore, the study examined the number of patients in both groups who achieved at least 30% and at least 50% pain relief. Adverse events that occurred during the treatment period were recorded on a daily basis. All scores, including pain assessment and QOL measures, were evaluated both before (T0) and one week after the completion of the treatment (T3).

### 2.5. Estimation of Sample Size

Sample size estimation was based on convenience sampling. The study specifically focused on individuals present at the Department of Pain Research and Treatment of the Jagiellonian University Medical College in Krakow, Poland between 20 January 2021, and 30 August 2022, who met the inclusion criteria and provided consent to participate in the study. Every eligible individual who was present during this period and agreed to participate was included in the study, resulting in the inclusion of the maximum number of participants possible.

### 2.6. Statistical Analysis

Distributions of quantitative variables were summarized with mean, standard deviation, median, and quartiles; whereas distributions of qualitative variables were summarized with the number and percent of occurrence for each of their values. The chi-squared test (with Yates’ correction for 2 × 2 tables) was used to compare qualitative variables among groups. In the case of low values in contingency tables, Fisher’s exact test was used instead. The Mann–Whitney test was used to compare quantitative variables between two groups, while the Kruskal–Wallis’s test (followed by the Dunn post-hoc test) was used for more than two groups. The relationship between two quantitative variables was assessed with Spearman’s coefficient of correlation. A paired Wilcoxon test was used to compare two repeated measures of quantitative variables. The significance level for all statistical tests was set to 0.05. R 4.3.1 was used for computations [25]. Included graphs show medians with the 95% confidence intervals.

## 3. Results

### 3.1. Baseline Group Characteristics

The characteristics of the patients are outlined in Table 1. The study groups were comparable and demonstrated homogeneous baseline characteristics, ensuring comparability between them. In the context of this study, all included patients self-identified with their respective gender and sex categories, ensuring accurate representation and understanding of their unique healthcare needs. No significant differences were observed between males and females in both study groups with regard to their characteristics at the initial assessment. The NRSa, NRSm, and NRSe scores before treatment did not exhibit any significant differences between the groups (see Table 2). However, a notable difference was observed in the Bodily Pain (BP) domain of the SF-36 questionnaire, with higher scores observed in the MA group. No significant differences were found in the other domains of the SF-36 questionnaire between the groups at the study’s outset (see Table 3).

#### 3.1.1. Comparative Analysis of Average NRS Pain Scores Pre- and Post-Treatment

**Figure 4:**
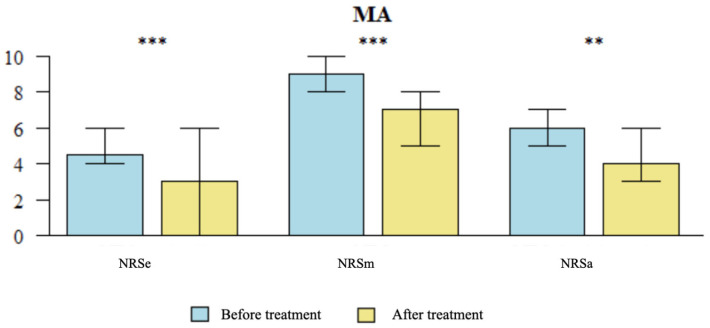
Comparison of NRS Pain Scores Pre- and Post-Treatment in MA group NRS: Numeric Rating Scale; NRSe: NRS score at the time of examination; NRSm: maximum NRS score in the past week; NRSa: average pain experienced by the patients over the previous week. MA: Medical acupuncture. Statistically significant difference (*p* < 0.05); ** *p* < 0.01; *** *p* < 0.001

**Figure 5:**
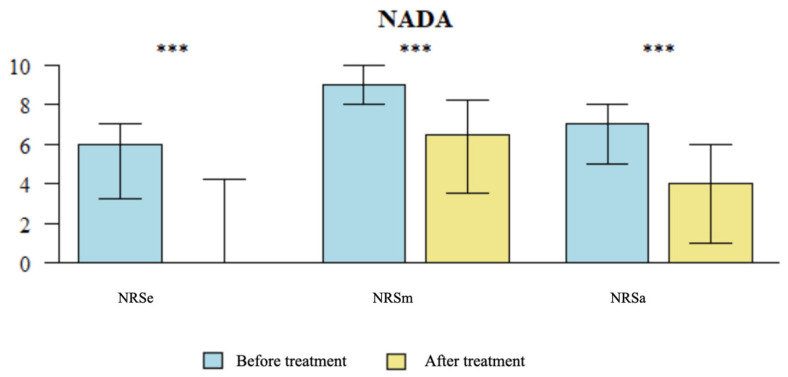
Comparison of NRS Pain Scores Pre- and Post-Treatment in NADA group NRS: Numeric Rating Scale; NRSe: NRS score at the time of examination; NRSm: maximum NRS score in the past week; NRSa: average pain experienced by the patients over the previous week. NADA: National Acupuncture Detoxification Association. Statistically significant difference (*p* < 0.05), *** *p* < 0.001

A significant reduction was observed in the NRSa score (*p* = 0.002) following treatment with MA (see Figure 4) compared to baseline (T0). Similarly, a significant decrease was found in the NRSa score (*p* < 0.001) compared to baseline (T0) after the utilization of NADA ear acupuncture treatment (see Figure 5). Notably, the NRSa score reduction was significantly greater in the NADA group (*p* = 0.034) (see Table 4).

#### 3.1.2. Comparative Analysis of Maximum NRS Scores Pre- and Post-Treatment

A significant reduction was observed in the NRSm score (*p* < 0.001) compared to baseline (T0) following treatment with MA (see Figure 4). Similarly, a significant decrease was found in the NRSm score (*p* < 0.001) compared to baseline (T0) after the utilization of NADA ear acupuncture treatment (see Figure 5). There were no significant differences between the two groups in the NRSm score reduction (see Table 4).

#### 3.1.3. Comparative Analysis of NRS Scores at the Time of Examination Pre- and Post-Treatment

A significant reduction was observed in the NRSe score (*p* = 0.001) compared to baseline following treatment with MA (see Figure 4). Similarly, a significant decrease was found in the NRSe score (*p* < 0.001) compared to baseline (T0) after the utilization of NADA ear acupuncture treatment (see Figure 5). There were no significant differences between the two groups in the reduction of the NRSe score (see Table 4).

#### 3.1.4. Efficacy Outcomes: Proportions of Patients Achieving 30% and 50% Reduction in Average NRS Pain Scores over the Previous Week

In terms of percentage pain decrease, it was observed that 9 (30%) patients in the MA group and 1 (3.33%) patient in the NADA group achieved a 30% reduction in the NRSa score compared to baseline (T0). Additionally, a 50% or greater decrease in the NRSa score was observed in 6 (20%) patients in the MA group and 14 (46.67%) patients in the NADA group compared to baseline (T0), indicating significantly better outcome in patients receiving NADA acupuncture (*p* = 0.007) compared to those receiving MA (see Table 5).

#### 3.1.5. Rescue Medication Utilization and Comparative Analysis among Study Cohorts

We conducted a comparison of rescue medication use between the 2 groups. In the MA group, patients statistically used 0.17 rescue medication tablets/day and in the NADA group, the rescue medication utilization was 0.04 tablets/day. The results indicated no statistically significant difference in rescue medication usage between the two groups (see Table 6).

### 3.2. Evaluation of QOL Changes Using SF-36 Questionnaire: Pre- and Post-Treatment Comparison

**Figure 6:**
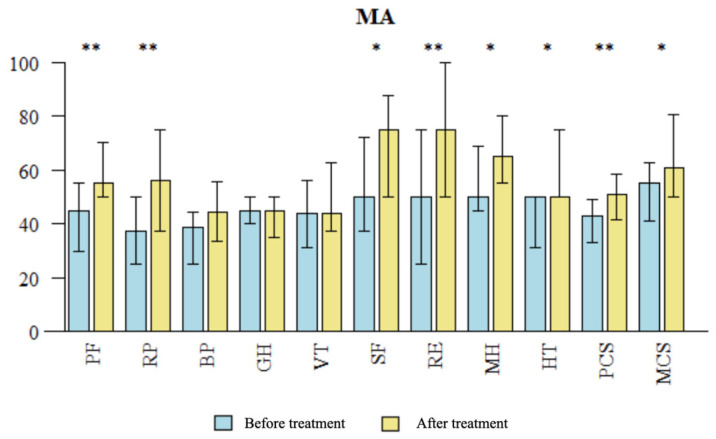
Comparison in QOL measures after treatment in the MA group The SF-36 questionnaire assesses quality of life (QOL) in 11 domains: PF: physical fitness; RP: role limitations due to physical problems; BP: bodily pain; GH: general health perception; VT: vitality; SF: social functioning; RE: role limitation due to emotional problems; MH: mental health; HT: health transition/change; PCS: total physical health; MCS: total mental health; PCS was calculated from RF, RP, BP and GH and MCS was calculated from VT, SF, RE, MH. MA: Medical acupuncture. * statistically significant difference (*p* < 0.05), ** *p* < 0.01.

**Figure 7:**
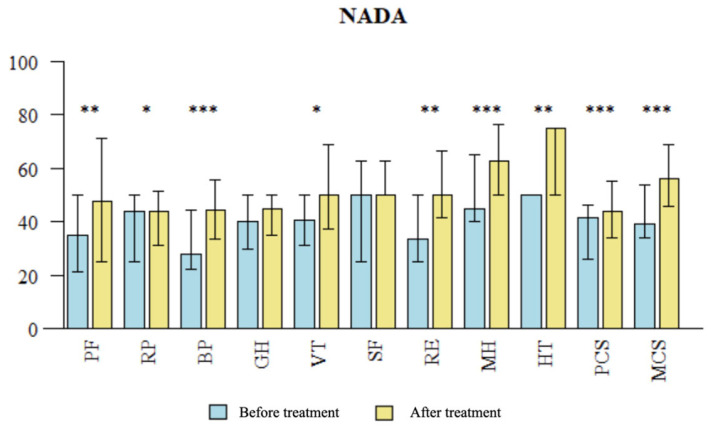
Comparison of QOL measures after treatment in the NADA group. The SF-36 questionnaire assesses quality of life (QOL) in 11 domains: PF: physical fitness; RP: role limitations due to physical problems; BP: bodily pain; GH: general health perception; VT: vitality; SF: social functioning; RE: role limitation due to emotional problems; MH: mental health; HT: health transition/change; PCS: total physical health; MCS: total mental health; PCS was calculated from RF, RP, BP and GH and MCS was calculated from VT, SF, RE, MH. NADA: National Acupuncture Detoxification Association. * statistically significant difference (*p* < 0.05); ** *p* < 0.01; *** *p* < 0.001.

Following treatment in the MA group, significant improvements were observed in the QOL of patients compared to baseline (T0) across several domains in the SF-36 questionnaire, including PF (*p* = 0.003), RP (*p* = 0.002), SF (*p* = 0.041), RE (*p* = 0.005), MH (*p* = 0.043), HT (*p* = 0.013), PCS (*p* = 0.004), and MCS (*p* = 0.025) (see Figure 6). There was observed improvement in the BP domain in the MA group, but it was not significant (*p* = 0.198). This might be due to initially (T0) higher scores in the BP domain in this group. Following NADA treatment, significant improvements in the QOL of patients compared to baseline were observed in the NADA group across various domains of the SF-36 questionnaire, including PF (*p* = 0.004), RP (*p* = 0.048), BP (*p* = 0.001), VT (*p* = 0.035), RE (*p* = 0.006), MH (*p* < 0.001), HT (*p* = 0.003), PCS (*p* < 0.001), and MCS (*p* < 0.001) (see Figure 7). There were no significant differences observed in the improvement of SF-36 domains compared to baseline between the two treatment modalities (see Table 7).

### 3.3. Adverse Events (AE) Analysis

There were minor complications observed in 35% of patients (total of 20 participants); 31% (9 patients) in the MA group and 39% (11 patients) in the NADA group (see Table 8). These AE were minor and quickly resolved, including insertion point pain, minor bleeding after needle removal, and one instance of fainting. No patients in either group reported worsening of LBP. These complications occurred in 4.14% of MA sessions (12 times/290 sessions) and in 6.07% NADA acupuncture sessions (16 times/280 sessions) (see Table 9). There were no statistically significant differences between the groups in the number of AE.

## 4. Discussion

The outcomes of this study suggest that both MA and NADA ear acupuncture could be a valuable component of a comprehensive approach to managing chronic nonspecific LBP in older patients. Incorporation of MA and NADA ear acupuncture into the clinical management of chronic nonspecific LBP in elderly patients has the potential to reduce pain intensity and improve the overall quality of life for affected individuals. When comparing the application of medical acupuncture to ear acupuncture according to the NADA protocol in older patients with chronic nonspecific LBP, we showed that neither method was superior in reducing NRS pain scores and improving QOL. We were the first to conduct a comparative evaluation of NADA ear acupuncture and MA as interventions for older individuals experiencing chronic nonspecific LBP.

Consistent with our research, analogous outcomes have been observed in previous studies for MA in the context of this condition. Numerous clinical trials have extensively investigated the efficacy of acupuncture as a treatment for chronic nonspecific LBP. A recent meta-analysis conducted in 2020, which encompassed a total of 8270 patients, demonstrated that acupuncture yielded significant and clinically meaningful pain relief, as well as improved back function in the short term when compared to no treatment (supported by moderate-certainty evidence), however the mean age of the participants was 41.6 years [26]. A previous systematic review and meta-analysis conducted in 2017 in Iran revealed that acupuncture, acupressure, and chiropractic interventions may have favorable effects on self-reported pain and functional limitations in non-pregnant adults (>15 years of age) with chronic nonspecific LBP [27]. Furthermore, a randomized controlled trial comparing the effectiveness of manual and electrical acupuncture, involving 66 patients with chronic nonspecific LBP aged between 20 and 60 years, revealed that both therapies exhibited comparable efficacy in reducing pain intensity measured on the NRS scale, as well as alleviating disability as measured by the Roland Morris Disability Questionnaire [28]. When considering the reduction in pain intensity, our study’s findings are consistent with these results; however, in contrast to our study, the researchers [28] did not study the effectiveness in elderly patients.

While numerous studies have investigated the effects of MA on chronic LBP, to date, there are no data available referring to the examination of the NADA ear acupuncture protocol for chronic nonspecific LBP. One study partially implemented the NADA protocol by utilizing two points, “Shenmen” and “Kidney”, in addition to the reflex point in the ear corresponding to the lumbar or sacral regions. This was a multicenter randomized trial investigating the effects of ear acupuncture on pregnancy-related pain in the lower back and posterior pelvic girdle (LBPGP) [29]. It was found that after a two-week treatment period, ear acupuncture administered by midwives alongside standard obstetric care resulted in a significant reduction in lumbar and pelvic pain in pregnant women, as well as improvements in their QOL and reduction in functional disability compared to the sham acupuncture group [29]. Drawing direct comparisons between the outcomes of our study and the aforementioned study is not possible, given significant disparities in study cohorts and variations in the utilized acupuncture protocol.

Another study using the hand-ear acupuncture and standard acupuncture modes concluded that both methods have beneficial and persistent effectiveness against chronic LBP compared with the usual care [30]. Furthermore, hand-ear acupuncture was significantly more effective than standardized acupuncture, especially in the long term [30]. In this study, only one ear point (Yaotongdian (AH 9)) and one hand acupuncture point (Yaotongdian (EX-UE 7)) were used [30]. Direct comparisons between the findings of our study and those of the aforementioned study are not feasible due to notable dissimilarities in the study cohorts and variations in the employed acupuncture protocol.

After conducting a thorough search, we identified only one study that investigated the application of the NADA ear acupuncture protocol in assessing QOL according to the SF-36 questionnaire among older adults suffering from depression [31]. However, this study did not include an assessment of pain [31]. The German study of Geib et al. involved 20 psychogeriatric patients with major depression attending a daytime clinic and showed high acceptance of NADA ear acupuncture among all participants [31]. Authors reported significant improvements in depression scores and in the QOL (SF-36 questionnaire) compared to the control group [31]. The patients’ dominant perception was a positive expectation and conviction that acupuncture was an effective form of therapy for depression without side effects, which played a role in their recovery [31]. The observed improvement in the QOL in this study aligns with our own findings; however, there are several differences between our study and the German study [31]. First of all, the German study examined the NADA protocol only in depression, whereas we were assessing QOL in elderly patients suffering from pain. It is noteworthy to mention that in our study, patients in the MA group presented a statistically significant higher quality of life in the BP domain of the SF-36 questionnaire prior to the intervention compared to the NADA group. Consequently, the possibility of selection bias and the impact of non-randomization cannot be ruled out. It is plausible that patients with a lower initial quality of life due to pain (BP SF-36 domain) may have been more inclined to explore a “new method” of pain treatment (NADA ear acupuncture).

The study of Geib et al. mentioned the importance of patients’ expectations on the outcome of the treatment. The role of patients’ expectations in LBP represents an emerging field of investigation [32]. From a clinical perspective, patients’ expectations represent conscious phenomena that influence their response to treatment in various musculoskeletal conditions, including chronic LBP [32]. In the narrative review on exploring the role expectations have on the results of the treatment of patients with chronic LBP, Ballestra et al. showed that certain patients’ expectations were significantly associated with better recovery outcomes in a conservative treatment of working-age patients with chronic LBP [32]. The studied expectations included expectation of a tailored exercise program with frequent follow-ups, the hope for the best possible outcomes, realism or resignation regarding pain relief, activity levels, good dialogue and communication, the need to be seen and confirmed as an individual, and the desire to receive an explanation and education about their pain [32]. Moreover, the expectation of results might have been highly influenced by patients’ previous experiences [32]. The results of Ballestra et al. are consistent with the biopsychosocial model of pain, in which pain is the result of complex interactions between biological, psychological, and sociological factors that individuals experience and individuals’ treatment approach should take all the elements under consideration [33]. Steven George, during a workshop on the role of nonpharmacological approaches to pain management, described pain using the analogy of an onion, with the layers being nociception, individual experience such as the beliefs, emotions, and coping strategies, and impact that pain has on the person. The impact of pain on a person’s life was dependent on the experience [6]. An observational study with 5 years of follow-up that involved 281 patients showed that negative illness perceptions (negative cognitive and emotional responses to LBP) may contribute to higher risks of a persistent pain trajectory among patients with nonspecific LBP with a high pain intensity [34]; it might also affect treatment outcomes. For example, Higa et al. suggested that people with high-level catastrophic thinking may find it difficult to obtain the analgesic effects of electroacupuncture [35].

In our study we did not assess patients’ expectations and we are unable to exclude their influence on the results of the treatment. However, we can assume that the patients’ expectations might have played a role in their recovery. Referring to Ballestra et al. we may distinguish some expectations that might have appeared among the patients of our study, like the expectations towards the clinical professional, which include the desire to receive information and education about the disorder, to obtain knowledge about the content of treatment, the treatment setting, attention and interest in their clinical course; along with extensive knowledge of the body and pain on the part of the health care professional, and the expectation of some type of physical examination. The initial examination of the patient in this study lasted between 1–1.5 h, which presents a much greater amount of time than a usual visit at the Department for Pain Research and Treatment. Patients were able to receive comprehensive and detailed information on the treatment modalities used in the study (MA and NADA). Their quality of life was also assessed in a very comprehensive way using the SF-36 questionnaire. The patients probably also felt more cared for as they knew that they were participating in a study and all their complaints were noted, and they have been constantly under the supervision of the study team. Regarding the recruitment process, it is not possible to exclude the expectation factor on the patients’ decision to join the NADA ear acupuncture group; the patients might have expected that the “new” modality of LBP treatment would work for them.

As suggested before, expectations may play a role in the outcomes of the treatment; in the study of Sánchez Romero et al., it appears that this did not apply to the expectations induced by verbal suggestions [36]. Sánchez Romero et al. conducted a randomized controlled trial including healthy subjects who were randomly assigned to one of three groups receiving different verbal suggestions about the effects of dry needling and the occurrence of post-needling soreness (positive, negative, or neutral) [36]. The authors of this RCT found that the induction of different types of expectations through verbal suggestion did not influence the perception of acute pain perceived during the performance of a deep dry-needling technique and post-needling pain or soreness after deep dry needling on a latent upper trapezius myofascial trigger point (MTrP) [36]. According to the study, there were no differences between the groups on the intensity of post-needling soreness or tenderness over a one-week follow-up [36]. Moreover, verbal suggestion was not associated with changes in sensorimotor variables of temporal summation (TS) and conditioned pain modulation (CPM) [36]. However, the authors admitted that they did not directly assess the patients’ own beliefs or expectations about post-needling soreness or about the deep dry-needling technique to see how the patients’ expectations were induced by the therapist’s verbal suggestion, which they considered a limitation of their study [36]. Previous research revealed that verbal suggestion interventions to induce analgesic expectations relieved patients’ procedural pain, suggesting they could be used to optimize the effectiveness of standard analgesic treatments in clinical practice [37]. On the other hand, inducing negative expectations regarding adverse effects, such as verbal suggestions of potential side effects may lead to the experience of aversive side effects [38]. In our study we tried to avoid any type of verbal suggestions. The patients were given written information, including a description of the study and methods used; they received information on the possible side effects of the acupuncture treatment as well.

Referring to Ballestra et al., future studies should aim to evaluate patients’ expectations before and after treatment administration and analyze the prognostic value of patients’ expectations in chronic nonspecific LBP [32]. It would also be beneficial to analyze how expectations develop in patients with chronic nonspecific LBP and to evaluate them before and after applying a treatment within an evidence-based approach.

While the mechanisms of action of acupuncture have been extensively studied over the past years, more recent research has enabled a broader understanding of the underlying complex mechanisms of auriculotherapy [17]. Newer theoretical perspectives have highlighted the role of the vagus nerve and its regulation by the hypothalamus of the brain [17]. While inflammation may be one of the mechanisms underlying chronic LBP, patients with chronic LBP may potentially benefit from the vagus nerve role in the anti-inflammatory pathway. This anti-inflammatory action of the vagus nerve has been the subject of extensive research conducted by Tracey and his colleagues [39]. In one of their seminal papers, they emphasized the importance of regulating the innate immune response as a crucial factor in controlling inflammation and the prevention and treatment of diseases [39]. The efferent vagus nerve is involved in inhibiting the release of pro-inflammatory cytokines and providing protection against systemic inflammation in the “cholinergic anti-inflammatory pathway” [39]. The ear is innervated by both the sympathetic and parasympathetic divisions of the autonomic nervous system (ANS). The sympathetic innervation of the ear arises from the superior cervical ganglion and travels to the ear via the carotid plexus [40]. The parasympathetic innervation of the ear arises from the glossopharyngeal and vagus nerves and travels to the ear via the auricular branch of the vagus nerve [40]. The complex interplay between sympathetic and parasympathetic innervation in the ear has an important role in regulating various physiological processes and may contribute to the therapeutic effects of auricular acupuncture [17]. By stimulating specific ear acupuncture points, it is possible to modulate the activity of the ANS and influence the regulation of various bodily functions, including the perception of pain [17]. For example, acupuncture at the “Shenmen” ear point was shown to slow down the heart rate and activate parasympathetic nerves in humans [41]. In the future investigations, it would be interesting to explore further the correlation between the autonomic nervous system, acupuncture and regulation of pain perception.

### 4.1. Clinical Implications

Integration of Acupuncture in Multimodal Pain Management Plans: The findings of this study, investigating MA and NADA ear acupuncture efficacy in the management of chronic nonspecific LBP in elderly patients, offer meaningful insights that can guide clinical decision making and potentially enhance patient care. In our opinion, in clinical practice, older patients with chronic, nonspecific LBP may benefit from MA and NADA ear acupuncture. Although more research is needed to confirm our findings, we believe that both MA and NADA ear acupuncture may become a valuable addition to multimodal treatment plans for elderly patients with chronic nonspecific LBP, offering patients a broader spectrum of nonpharmacologic pain relief options.

Cost-Effectiveness Considerations: Acupuncture, including ear acupuncture, may not only be effective in treating chronic LBP among older adults, but it also offers potential cost-reduction avenues [42]. Witt C.M. et al. reported in their study that acupuncture combined with routine care was associated with marked clinical improvements in patients with chronic LBP and was relatively cost-effective [42]. The Veterans Administration and various branches of the U.S. Military have already successfully utilized acupuncture, with some studies demonstrating decreased opioid prescriptions when included in care [43]. Acupuncture is suggested to be potentially safely, easily, and cost-effectively incorporated into various hospital settings to treat commonly seen pain conditions [43]. As healthcare systems worldwide seek cost-effective approaches to chronic pain management, it would be valuable to explore the potential cost-effectiveness of acupuncture. In the future, assessing the economic impact, including the potential reduction in healthcare utilization and medication costs, might be a good way to inform health policymakers and healthcare providers about the broader implications of integrating acupuncture into standard treatment protocols for chronic nonspecific LBP in older patients. With the increasing number of therapies available to patients with chronic lower back pain, future studies should consider cost-efficacy, as treatment can last for many years.

### 4.2. Study Considerations: Limitations, Strengths, and Implications

Our study had several limitations. First, the study design lacked randomization, and both the acupuncture providers and patients were not blinded to the treatment they received. Blinding in acupuncture studies can be challenging, especially when comparing different techniques or protocols. Furthermore, the absence of a control group limits the strength of the evidence. However, it is important to note that our study aimed to compare the effectiveness of two acupuncture protocols for chronic nonspecific LBP. Another potential limitation of the study is the inclusion of patients who have not received acupuncture treatment in the past three months only. This means that some patients may have had acupuncture more than three months prior to their inclusion and had chosen to participate in the study because they had a positive perception of acupuncture and found it beneficial for their condition. However, considering the complex nature of chronic pain and the importance of patient preferences, we believe that incorporating patients’ preferences into the study design is a valuable approach. Assignment to the NADA group might contribute to the selection of patients who were willing to try a new treatment due to the higher burden that pain had on their life (significantly greater score in the BP domain of SF-36 compared to MA group). Furthermore, the limited sample size in our study may reduce statistical power and limit the generalizability of the findings, and the demographic homogeneity of the study population may limit the generalizability of the findings to more a diverse population. Additionally, the variability in acupuncture techniques, with different acupuncturists employing varying techniques, needle placements, and treatment durations, introduces heterogeneity and hinders direct comparisons with other studies. However, it is worth noting that the NADA ear acupuncture protocol used in our study is one of the few standardized ear acupuncture protocols available. Though there is not a standardized protocol for acupuncture in LBP, our pain department developed this MA protocol using common locations for chronic LBP that is easy to reproduce. Another limitation of this study is the potential placebo effect of acupuncture, which can complicate the differentiation between specific and nonspecific effects. Nonetheless, as our study focused on comparing two acupuncture protocols rather than acupuncture against other treatments, we believe that the influence of placebo effects on our results may be limited. What is more, the data from the literature indicate that the placebo response correlates negatively with increasing patients’ age [44]. Another limitation is that the study lacked long-term follow-up, resulting in a relatively short duration of observation, which makes it challenging to assess the durability of treatment effects over the long term. In our study we also did not use the standard core outcome set (COS) [45]. In 2015, Chiarotto et al. updated the COS through an international and multidisciplinary consensus called the Delphi study [45]. This updated COS for nonspecific LBP included the following core outcome domains: physical functioning, pain intensity, health-related quality of life (HRQOL), and number of deaths [45]. It is hard to directly compare the results of our study with other studies on nonspecific LBP due to the great heterogeneity still existing in the measurement instruments used. Innocenti et al. stated that although the recommended core outcome set (COS) for studies on nonspecific LBP has been in the public domain for over 20 years, only a small proportion (20.8%) of studies aimed to measure all COS domains, and the COS uptake is not increasing over time [46]. Inclusion of patients with nonspecific LBP only limits the generalizability of our results on other patients with chronic LBP, like specific LBP. Different sizes of needles (0.30 mm × 30 mm vs. 0.16 mm × 15 mm) were used in this study for NADA and MA, which can make it difficult to directly compare the two protocols, although these were standard needles used for these parts of the body. Lastly, various sources of bias, such as selection bias or publication bias, may impact the validity and generalizability of our study’s results.

The advantages of this study were in acquiring a comprehensive insight into the distinctions between NADA ear acupuncture and MA treatments. Both acupuncture methods may become a viable, safe, easily applicable, and well-received alternative for managing chronic nonspecific LBP in older adults. Furthermore, this study aimed to provide a practical framework for clinical decision making in this domain, thereby augmenting patient-centered care by accommodating preferences and consequently enhancing overall treatment outcomes. Additionally, the study aimed to empower older adults by providing them with informed healthcare choices and to chart a course for future endeavors in nonpharmacological pain management strategies. This was also the first study to assess and compare the MA and NADA ear acupuncture protocol in older patients with chronic nonspecific LBP.

## 5. Conclusions

In summary, the outcomes of this study suggest that both MA and NADA ear acupuncture could be valuable components of a comprehensive approach to managing chronic nonspecific LBP in older patients. The incorporation of MA and NADA ear acupuncture into the clinical management of chronic nonspecific LBP in elderly patients has the potential to reduce pain intensity and improve the overall quality of life for affected individuals. However, further studies are needed to confirm our findings. Standardization of acupuncture protocols and use of the core outcome set in the studies of chronic nonspecific LBP pain may play a beneficial role in interpreting results from future studies.

## Figures and Tables

**Table 1:** Baseline characteristics of the subjects by group.

		MA Group (*n=* 30)	NADA Group (*n* = 30)	Total (*n* = 60)	*p*
Age [years]	Mean (SD)	71.13 (8.64)	71.57 (7.3)	71.35 (7.93)	*p* = 0.695
	Median (quartiles)	69.5 (64–78.25)	70 (65.25–77.5)	70 (64–78)	
	Range	60–94	60–87	60–94	
Gender	Female	22 (73.33%)	24 (80.00%)	46 (76.67%)	*p* = 0.76
	Male	8 (26.67%)	6 (20.00%)	14 (23.33%)	
BMI [kg/m²]	Mean (SD)	26.87 (3.62)	28.25 (5.89)	27.56 (4.9)	*p* = 0.515
	Median (quartiles)	27.18 (25.14–29)	27.95 (23.77–31.07)	27.33 (24.43–30.41)	
	Range	17.97–34.08	18.82–42.22	17.97–42.22	
BMI	Healthy weight	8 (26.67%)	10 (33.33%)	18 (30.00%)	*p* = 0.417
	Overweight	15 (50.00%)	10 (33.33%)	25 (41.67%)	
	Obesity	7 (23.33%)	10 (33.33%)	17 (28.33%)	

Qualitative variables: chi-squared test or Fisher’s exact test; Quantitative variables: Mann–Whitney test, BMI: body mass index. *n*: number of patients.

**Table 2:** NRS comparisons between groups before treatment.

	Group	*n*	Mean	SD	Median	Min	Max	Q1	Q3	*p*
**NRSe**	MA	30	4.90	2.70	4.5	0	10	4.00	6	*p* = 0.512
	NADA	30	5.17	2.95	6.0	0	10	3.25	7	
**NRSm**	MA	30	8.67	1.71	9.0	4	10	8.00	10	*p* = 0.521
	NADA	30	8.47	1.66	9.0	4	10	8.00	10	
**NRSa**	MA	30	6.07	1.64	6.0	3	9	5.00	7	*p* = 0.208
	NADA	30	6.67	1.79	7.0	4	10	5.00	8	

NRS: Numeric Rating Scale; NRSe: NRS score at the time of examination; NRSm: maximum NRS score in the past week; NRSa: average pain experienced by the patients over the previous week. *p*: Mann–Whitney test; SD: standard deviation; Q1: lower quartile; Q3: upper quartile; *n*: number of patients. The NRS pain severity scores range from 0 to 10, with higher scores indicating worse pain.

**Table 3:** SF-36 QOL comparisons between groups before treatments at T0.

	Group	*n*	Mean	SD	Median	Min	Max	Q1	Q3	*p*
**PF**	MA	30	41.83	20.61	45.00	0.00	75.00	30.00	55.00	*p* = 0.292
	NADA	30	36.83	21.48	35.00	0.00	85.00	21.25	50.00	
**RP**	MA	30	38.12	18.74	37.50	0.00	75.00	25.00	50.00	*p* = 0.935
	NADA	30	39.17	20.82	43.75	0.00	75.00	25.00	50.00	
**BP**	MA	30	40.37	19.68	38.89	0.00	100.00	25.00	44.44	*p* = 0.043 *
	NADA	30	30.37	17.73	27.78	0.00	77.78	22.22	44.44	
**GH**	MA	30	44.50	10.61	45.00	25.00	80.00	40.00	50.00	*p* = 0.173
	NADA	29	39.48	12.63	40.00	20.00	60.00	30.00	50.00	
**VT**	MA	29	45.47	18.59	43.75	0.00	75.00	31.25	56.25	*p* = 0.288
	NADA	30	41.67	18.59	40.62	12.50	100.00	31.25	50.00	
**SF**	MA	30	55.42	22.43	50.00	0.00	100.00	37.50	71.88	*p* = 0.152
	NADA	30	45.42	24.89	50.00	0.00	100.00	25.00	62.50	
**RE**	MA	30	51.94	33.09	50.00	0.00	100.00	25.00	75.00	*p* = 0.276
	NADA	29	43.68	26.69	33.33	0.00	100.00	25.00	50.00	
**MH**	MA	30	54.83	17.83	50.00	20.00	100.00	45.00	68.75	*p* = 0.24
	NADA	30	50.50	19.40	45.00	20.00	90.00	40.00	65.00	
**HT**	MA	30	46.67	23.43	50.00	0.00	100.00	31.25	50.00	*p* = 0.557
	NADA	30	50.00	18.57	50.00	0.00	75.00	50.00	50.00	
**PCS**	MA	30	41.54	10.62	43.08	20.00	60.00	33.08	48.85	*p* = 0.179
	NADA	29	37.24	14.01	41.54	9.23	67.69	26.15	46.15	
**MCS**	MA	29	51.42	16.96	55.36	10.71	76.79	41.07	62.50	*p* = 0.08
	NADA	29	45.63	17.09	39.29	19.64	85.71	33.93	53.57	

The SF-36 questionnaire assesses quality of life (QOL) in 11 domains: PF: physical fitness; RP: role limitations due to physical problems; BP: bodily pain; GH: general health perception; VT: vitality; SF: social functioning; RE: role limitation due to emotional problems; MH: mental health; HT: health transition/change; PCS: total physical health; MCS: total mental health; PCS was calculated from RF, RP, BP and GH and MCS was calculated from VT, SF, RE, MH. T0—initial evaluation, before beginning of acupuncture sessions. *p*: Mann–Whitney test; SD: standard deviation; Q1: lower quartile; Q3: upper quartile; *n*: number of patients. * statistically significant difference (*p* < 0.05)

**Table 4:** Comparison of changes in NRS scores between MA and NADA group.

	Group	*n*	Mean	SD	Median	Min	Max	Q1	Q3	*p*
**∆ NRSe**	MA	29	2.00	2.79	2	−2	10	0	3	*p* = 0.333
	NADA	28	2.96	3.44	2	−3	10	0	6	
**∆ NRSm**	MA	29	2.48	2.37	2	−2	10	1	4	*p* = 0.942
	NADA	28	2.93	3.18	2	−1	10	0	5	
**∆ NRSa**	MA	29	1.38	1.95	1	−2	5	0	3	*p* = 0.034 *
	NADA	28	2.71	2.54	3	−3	9	1	4	

NRS: Numeric Rating Scale; ∆NRSe: change in NRS score at the time of examination before and after treatment; ∆NRSm: change in maximum NRS score in the past week before and after treatment; ∆NRSa: change in average pain experienced by the patients over the previous week before and after treatment. MA: Medical acupuncture. NADA: National Acupuncture Detoxification Association. *p*: Mann–Whitney test; SD: standard deviation; Q1: lower quartile; Q3: upper quartile. * statistically significant difference (*p* < 0.05).

**Table 5:** Comparing change of pain relief based on NRS scores between groups.

	Relief in Pain	MA Group	NADA Group	*p*
**∆NRSe**	<30% or no relief	11 (36.67%)	10 (33.33%)	*p* = 0.135
	30–49%	4 (13.33%)	0 (0.00%)	
	>50% or more	12 (40.00%)	16 (53.33%)	
	Lack of data	3 (10.00%)	4 (13.33%)	
**∆NRSm**	<30% or no relief	16 (53.33%)	15 (50.00%)	*p* = 0.48
	30–49%	6 (20.00%)	3 (10.00%)	
	>50% or more	7 (23.33%)	10 (33.33%)	
	Lack of data	1 (3.33%)	2 (6.67%)	
**∆NRSa**	<30% or no relief	14 (46.67%)	13 (43.33%)	*p* = 0.007 **
	30–49%	9 (30.00%)	1 (3.33%)	
	>50% or more	6 (20.00%)	14 (46.67%)	
	Lack of data	1 (3.33%)	2 (6.67%)	

NRS: Numeric Rating Scale; ∆NRSe: change in NRS score at the time of examination before and after treatment; ∆NRSm: change in maximum NRS score in the past week before and after treatment; ∆NRSa: change in average pain experienced by the patients over the previous week before and after treatment. MA: Medical acupuncture. NADA: National Acupuncture Detoxification Association. *p*: chi-squared test or Fisher’s exact test. Statistically significant difference (*p* < 0.05), ** *p* < 0.01.

**Table 6:** Comparison of use of rescue medication between groups.

Group	*n*	Rescue Medication Use throughout Treatment							*p*
		Mean	SD	Median	Min	Max	Q1	Q3	
MA	29	1.66	2.72	0	0	10	0	2.00	*p* = 0.086
NADA	28	0.36	0.68	0	0	2	0	0.25	

MA: Medical acupuncture. NADA: National Acupuncture Detoxification Association. *p*: Mann–Whitney test; SD: standard deviation; Q1: lower quartile; Q3: upper quartile. *n*: number of patients Statistically significant difference (*p* < 0.05).

**Table 7:** Comparison of changes in QOL measures with treatment between groups.

	Group	*n*	Mean	SD	Median	Min	Max	Q1	Q3	*p*
∆PF	MA	29	10.00	17.98	15.00	−50.00	35.00	0.00	20.00	*p* = 0.574
	NADA	28	10.89	15.81	5.00	−10.00	55.00	−5.00	20.00	
∆RP	MA	29	20.69	30.39	25.00	−50.00	81.25	−6.25	43.75	*p* = 0.054
	NADA	28	6.03	15.45	6.25	−25.00	50.00	0.00	12.50	
∆BP	MA	29	3.45	29.41	11.11	−88.89	55.56	0.00	22.22	*p* = 0.163
	NADA	28	15.08	20.56	11.11	−22.22	66.67	0.00	22.22	
∆GH	MA	29	−0.17	12.36	0.00	−25.00	30.00	−10.00	5.00	*p* = 0.409
	NADA	27	3.89	13.96	0.00	−15.00	40.00	−5.00	7.50	
∆VT	MA	28	2.46	19.35	6.25	−43.75	37.50	−6.25	12.50	*p* = 0.424
	NADA	28	6.47	17.14	6.25	−31.25	56.25	0.00	12.50	
∆SF	MA	29	10.78	27.29	12.50	−50.00	50.00	−12.50	37.50	*p* = 0.846
	NADA	28	10.71	29.99	12.50	−50.00	87.50	−3.12	25.00	
∆RE	MA	29	15.23	31.74	25.00	−75.00	91.67	0.00	25.00	*p* = 0.734
	NADA	27	12.96	23.27	8.33	−41.67	75.00	0.00	25.00	
∆MH	MA	29	6.90	20.63	10.00	−50.00	40.00	−5.00	20.00	*p* = 0.47
	NADA	28	11.79	12.85	15.00	−15.00	45.00	5.00	20.00	
∆HT	MA	29	13.79	25.52	0.00	−50.00	50.00	0.00	25.00	*p* = 0.953
	NADA	28	14.29	21.97	12.50	−25.00	75.00	0.00	25.00	
∆PCS	MA	29	8.59	13.50	6.15	−16.92	41.54	1.54	13.85	*p* = 0.915
	NADA	27	8.66	10.24	7.69	−6.15	47.69	3.85	12.31	
∆MCS	MA	29	7.97	17.96	8.04	−30.36	50.00	−0.89	17.86	*p* = 0.533
	NADA	27	10.58	12.87	8.93	−16.07	39.29	2.68	19.64	

The SF-36 questionnaire assesses quality of life (QOL) in 11 domains: ∆PF: change in physical fitness scores; ∆RP: change in role limitations due to physical problems scores; ∆BP: change in bodily pain scores; ∆GH: change in general health perception scores; ∆VT: change in vitality scores; ∆SF: change in social functioning scores; ∆RE: change in role limitation due to emotional problems scores; ∆MH: change in mental health scores; ∆HT: change in health transition/change scores; ∆PCS: change in total physical health scores; ∆MCS: change in total mental health scores. MA: Medical acupuncture. *p*: Mann–Whitney test; SD: standard deviation; Q1: lower quartile; Q3: upper quartile. *n*: number of patients.

**Table 8:** Summary of adverse events (AE) in both groups.

AE	Group		*p*
	MA (*n* = 29)	NADA *(n* = 28)	
Insertion point pain	3 (10.34%)	2 (7.14%)	*p* = 1
Minor bleeding after needle removal	5 (17.24%)	6 (21.43%)	*p* = 0.948
Worsening of LBP	0 (0.00%)	0 (0.00%)	*p* = 1
Worsening of headache	0 (0.00%)	1 (3.57%)	*p* = 0.491
Euphoria	0 (0.00%)	1 (3.57%)	*p* = 0.491
Symptomatic decrease of blood pressure after the treatment session with accompanying weakness	0 (0.00%)	1 (3.57%)	*p* = 0.491
Fainting	1 (3.45%)	0 (0.00%)	*p* = 1

*p*—chi-squared test or Fisher’s exact test. AE: adverse events; MA: Medical acupuncture; NADA: National Acupuncture Detoxification Association. *n*: number of patients. Statistically significant difference (*p* < 0.05).

**Table 9:** Summary of adverse events (AE), taking into account the number of acupuncture sessions in the MA and NADA groups.

AE	Group		*p*
	MA (*n* = 290)	NADA (*n* = 280)	
Insertion point pain	3 (1.03%)	3 (1.07%)	*p* = 1
Minor bleeding after needle removal	8 (2.76%)	9 (3.21%)	*p* = 0.941
Worsening of LBP	0 (0.00%)	0 (0.00%)	*p* = 1
Worsening of headache	0 (0.00%)	1 (0.36%)	*p* = 0.491
Euphoria	0 (0.00%)	2 (0.71%)	*p* = 0.241
Symptomatic decrease of blood pressure after the treatment session with accompanying weakness	0 (0.00%)	1 (0.36%)	*p* = 0.491
Fainting	1 (0.34%)	0 (0.00%)	*p* = 1

*p*—chi-squared test or Fisher’s exact test. AE: adverse events; MA: Medical acupuncture; NADA: National Acupuncture Detoxification Association. *n*: number of acupuncture sessions. Statistically significant difference (*p* < 0.05).

## Data Availability

The authors confirm that the data supporting the findings of this study are available within the article.
